# Night Work and Sustainable Working Life—A Prospective Trajectory Analysis of Swedish Twins

**DOI:** 10.3390/ijerph191710857

**Published:** 2022-08-31

**Authors:** Annina Ropponen, Mo Wang, Auriba Raza, Jurgita Narusyte, Pia Svedberg

**Affiliations:** 1 Division of Insurance Medicine, Department of Clinical Neuroscience, Karolinska Institutet, SE-171 77 Stockholm, Sweden; 2 Finnish Institute of Occupational Health, FI-00032 Työterveyslaitos, Finland; 3 Center for Epidemiology and Community Medicine, Stockholm County Council, SE-104 31 Stockholm, Sweden

**Keywords:** sick leave, sustainable work, night work, shift work, cohort study, prospective study

## Abstract

The aim was to investigate the changes in sustainable working life over 10–13 years of follow-up and the effect of baseline night work. Data from the Swedish national registers were used to define sustainable working life. Survey data in the 1998–2003 “SALT” with 34,680 twins or in the 2004–2006 “STAGE” with 19,637 twins were utilized to assess night work at baseline. Group-based trajectory and multinomial regression models were applied. The results of the SALT cohort yielded five trajectory solutions: stable sustainable working life (40%), stable lack of sustainable working life (25%), later decreasingly sustainable working life (15%), increasingly sustainable working life (14%), and early decreasingly sustainable working life (7%). In the STAGE cohort, four trajectories were detected: stable sustainable working life (83%), decreasingly sustainable working life (7%), stable lack of sustainable working life (5%), and increasing sustainable working life (5%). Night work was associated with the decreasing or increasing sustainable working life in the trajectory groups. To conclude, the largest parts of both cohorts followed trajectories of stable sustainable working lives. Night work was associated with both the trajectories of decreasing and increasing sustainable working lives.

## 1. Introduction

Shift work that includes night work is common due to provision of operations in many sectors, including services, security, and industry. Hence, both women and men work nights, and some occupations, such as security or night wards, may even require working primarily at night [1,2,3]. Many negative health outcomes have been linked with night work, such as depression [4,5], hypertension [6], cardiovascular health problems [7], many cancers [8,9,10], and metabolic syndrome [11]. On the other hand, night work has also been associated with disability pension (DP) [12,13]. The potential pathways from night work to impaired health and/or the subsequent effects on short- or long-term absences from working life in terms of sickness absence (SA) or DP [5,14] can be linked through psychosocial, behavioral, or physiological mechanisms [2,15]. Furthermore, night work affects sleep; hence, the mechanisms also include circadian dysrhythmia and disturbed sleep [2,15]. Thus, further knowledge should be gained on the long-term effects of night work on sustainable working life, i.e., not having periods of long-term SA, DP, or unemployment. 

Sustainable working life assumes an individual’s sustainable ability to work [16,17] and is important not only for individuals, but also for workplaces and societies due to the need to prolong working lives. Hence, there is a need to promote the fit between work and the individual characteristics or circumstances during the course of life [18]. In terms of sustainable working life, this means that both work, i.e., night work in this study, and the individual, i.e., the employee and his/her characteristics (such as occupation [19], socioeconomic status, or education [20], and family situation [21]), merit attention. 

An important aspect related to night work is the “healthy worker effect”, which assumes that those who do not tolerate or have difficulties with night work do not continue, but transfer to day work [22]. Another aspect influencing night work is age, i.e., older employees may wish to work more day shifts than nights [23]. In addition, age-related trends in the associations between night work and health have been identified [24,25]. Therefore, an assumption exists that younger and older employees might react differently to night work [26,27]. This might be especially evident in longitudinal settings where working nights may change due to seniority and possibilities of selecting more favorable working hours [28,29]. Hence, night work might play a role in a sustainable working life.

Until now, to the best of our knowledge, studies of night work have been focused on the “unsustainable” part of the working life, i.e., SA and DP [12,13,14,30], whereas longitudinal studies of sustainable working life are lacking. In addition, studies with genetically informative samples, e.g., twins, are scarce, but have provided an important understanding of the role of familial confounding (i.e., genetics and shared—mainly in childhood—environments) in the associations between night work and SA or DP as proxies of sustainable working life [12,13,31]. These previous studies have shown that familial confounding cannot be ruled out for the associations between night work and DP.

This study aimed to investigate the changes in sustainable working life over 10–13 years of follow-up by using a group-based trajectory analysis. We also aimed to investigate the effects of night work and various sociodemographic factors on trajectory group membership and to assess the effects of familial factors by using night work discordant twin pairs.

## 2. Materials and Methods

The data for this study were available from the Swedish Twin Project of Disability Pension and Sickness Absence (STODS), which included the twins identified in the Swedish Twin Registry (STR) who were born between 1925 and 1990, i.e., 119,907 twin individuals. In this study, we utilized the national register data included in the STODS from the Swedish Social Insurance Agency MiDAS database for SA and DP in the years 1994–2016. Furthermore, we used data from the LISA database from Statistic Sweden (SCB) for the years 1994–2014 to assess unemployment, old-age pension, and sociodemographic factors [32]. Sustainable working life was estimated through the main labor market status in each year of follow-up by using the following definitions: SA/DP (>180 days with SA or DP benefits from the Social Insurance Agency); unemployment (>180 days with unemployment benefits); old-age pension (more than half of yearly income from old-age pension); employment (i.e., in paid work and not fulfilling the criteria for SA/DP, unemployment, or old-age pension). For statistical analyses, these statuses were coded for each year, where employment = “1” and all other statuses were “0”. Emigration (from LISA) and death from were taken from the Cause of Death Register from the National Board of Health and Welfare and were assessed for censoring.

**Figure 1:**
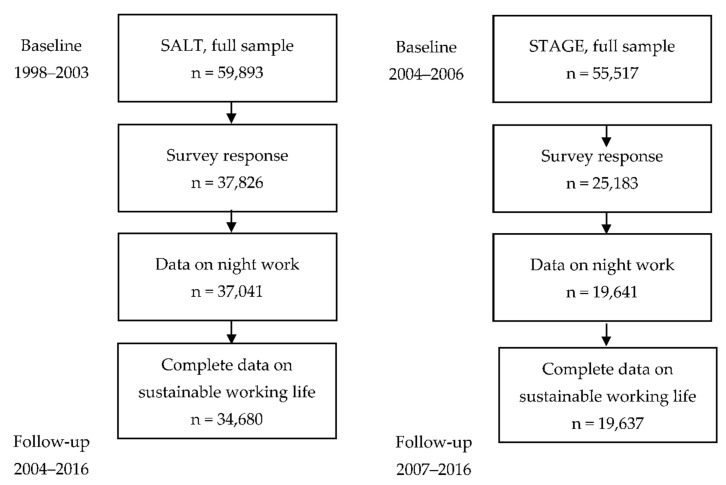
Sample definitions of the SALT and STAGE cohorts and their baseline and follow-up times.

The full sample with all register data for sustainable working life included 108,275 twin individuals. In addition to the register data, we utilized survey data collected by the Swedish Twin Registry (STR) from the SALT and STAGE surveys on night work. The SALT survey was conducted in 1998–2003, and the STAGE survey was conducted in 2005–2006. Hence, we analyzed the data in two cohorts, i.e., in the SALT cohort, we applied a follow-up from 2004 until 2016, and in the STAGE cohort, we applied a follow-up from 2007 until 2016; see Figure 1. The final sample for the SALT cohort, which is later also referred to as the “older cohort”, included 34,680 twin individuals born between 1929 and 1958, with complete data for night work and sustainable working life. Out of these, 3459 were complete monozygotic (MZ) pairs and 4723 were same-sexed dizygotic (DZ) pairs. The number of discordant MZ pairs involved in night work was 991, and there were 1707 DZ pairs. The final number of individuals in the STAGE cohort, who were born between 1959 and 1985 (later referred to as the younger cohort), with complete data for night work and sustainable working life was 19,637, which included 2296 complete MZ and 1733 same-sexed DZ pairs, of which 656 were discordant MZ pairs for baseline night work and 627 were DZ pairs.

The main exposure of interest was based on the self-reported night work (SALT and STAGE surveys) and was utilized in two ways. First, night work was evaluated with a dichotomized measure of “yes/no” concerning night work. The second measure used the following survey question: “For how many years have you had working hours that mean that you work nights at least now and then?”. We grouped the responses into three categories for night work exposure: not at all, 1–10 years, and >10 years, as has been done before [12,13]. We also included several sociodemographic factors for the prediction of belonging to the trajectory groups from the LISA data at baseline (i.e., at the time of the SALT and STAGE survey responses). The variables included were age, sex, marital status (married/living with someone vs. being single), education (at three levels based on years of education: 0–9, 10–12, and >12 years), and occupational sector (private, public, or other). 

## 3. Statistical Analyses

All statistical analyses were conducted with Stata 17.1 MP (Statacorp LLC., College Station, TX, USA). First, the descriptive statistics for the mean with standard deviation (SD) and frequencies with percentages (%) were calculated for sample characteristics, including sociodemographic characteristics and occupational sectors. The trajectories of the annual statuses of sustainable working life, i.e., employment, unemployment, SA/DP, and old-age pension, were estimated during the follow-up by using group-based trajectory modeling (GBTM) [33]. The GBTM method enables the identification of groups of individuals (trajectory groups) that follow a distinct pattern over time. The Bayesian information criterion (BIC), Akaike information criterion (AIC), average posterior probability (APP), and a priori assumption of ≥5% for the smallest group of trajectories were used to determine the best-fitting model. In addition, spaghetti plots (not shown) were visually inspected. A linear polynomial model was applied.

Then, multinomial regression models that included age, sex, marital status, education, night work, and occupational sector were estimated at the same time (i.e., in the same models) for each trajectory group. Two night work measures were separately analyzed in the full models of both cohorts. Relative-risk ratios (RRs) and 95% confidence intervals (CIs) were calculated. Conditional regression models were then run for night work discordant MZ and same-sexed DZ twin pairs to assess the presence of familial confounding. In the discordant twin pairs involved in night work, one twin within a twin pair had worked nights (based on the measure used) while the co-twin had not, providing us with a co-twin control design. This co-twin control analysis assumed that twins in a pair shared genes (100% in MZ pairs and, on average, 50% of their segregating genes in DZ twin pairs) and family background, that is, home and social family environment (mainly in childhood). The interpretation would be that familial confounding may not play a role if the association is found in the analyses of both MZ and DZ twins.

## 4. Results

The individuals in the SALT cohort had a mean age of 59 years (SD 7.8) at baseline, whereas the mean age in the STAGE cohort was 37 years (SD 7.1, Table 1). Furthermore, due to differences in the baseline years, the follow-up was 13 years for SALT and 10 years for STAGE. The sociodemographic factors are presented in Table 1.

Five trajectory solutions were chosen for the SALT cohort (Table 2), whereas four trajectories of sustainable working life were identified for the STAGE cohort.

**Figure 2:**
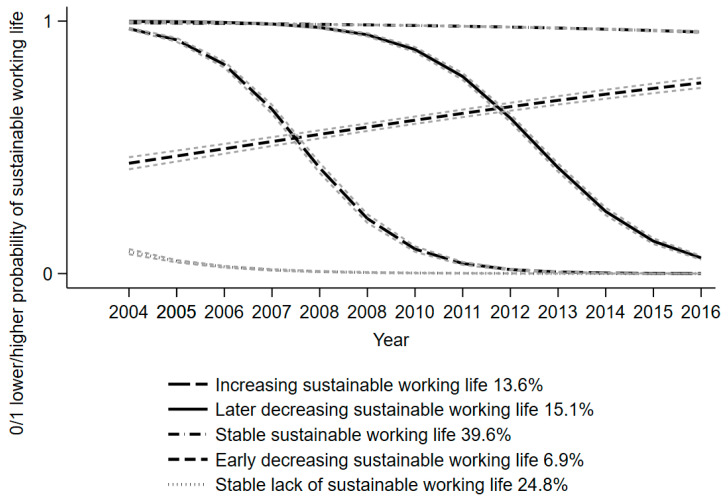
Trajectories of sustainable working life in the SALT cohort from 2004 to 2016. In the figure, some of the 95% confidence intervals are very narrow and almost invisible.

For the SALT cohort, the trajectory groups (Figure 2) were:

“stable sustainable working life” (39.6%), 

“stable lack of sustainable working life” (24.8%), 

“later decreasingly sustainable working life” (15.0%), 

“increasingly sustainable working life” (13.6%), and

“early decreasingly sustainable working life” (6.9%).

**Figure 3:**
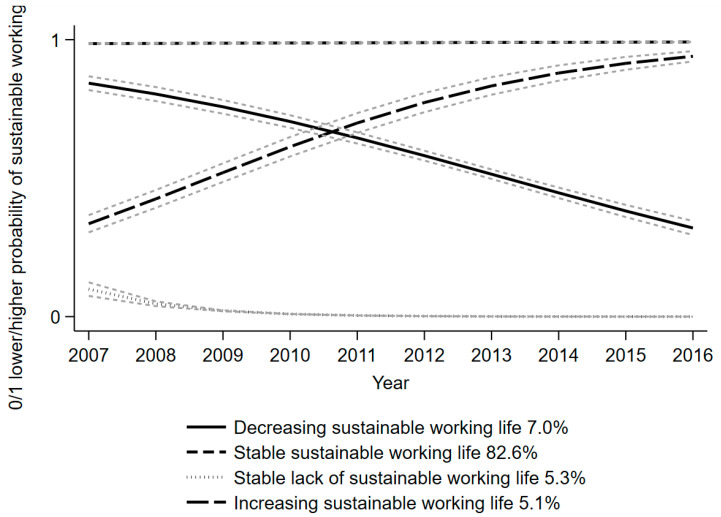
Trajectories of sustainable working life in the STAGE cohort from 2007 to 2016. In the figure, some of the 95% confidence intervals are very narrow and are almost invisible.

In the STAGE cohort, the trajectory groups (Figure 3) were:

“stable sustainable working life” (82.6%),

“decreasingly sustainable working life” (7.0%),

“stable lack of sustainable working life” (5.3%), and

“increasingly sustainable working life” (5.1%).

The Supplementary Material (Supplementary Table S2) shows that, in a stratified analysis of the SALT cohort, both those with and those without baseline night work had five trajectory group solutions at best. As shown in Supplementary Figures S1 and S2, the trajectories were the same as those detected in the analysis of the whole cohort.

The investigation of the factors that were influential for belonging to the other trajectory groups than the stable sustainable working life in the SALT cohort (Table 3) indicated that night work, which was measured either as “yes” or as a history of night work, was associated with a lower risk of belonging to the groups for later decreasingly sustainable working life or an increasing probability of sustainable working life in comparison to a stable highly sustainable working life. Each one-year increase in age predicted a lower likelihood of belonging to the group for a stable lack of sustainable working life, later decreasingly sustainable working life, and early decreasingly sustainable working life, whereas the opposite was observed for the group for increasingly sustainable working life. Being a woman (RR 1.17–1.46, Table 3, in comparison with being a man) or working in the private sector (RR 1.12–1.42) instead of the public sector predicted an increased likelihood of belonging to all of the trajectory groups (in comparison to the stable sustainable working life trajectory group). Higher education was linked with less likelihood (RR 0.57–0.86, Table 3) of belonging to all trajectory groups except the early decreasingly sustainable working life group. The analysis limited to discordant MZ and DZ twins involved in night work indicated that the associations retained their magnitude and direction, hence pointing towards there being no familial confounding in the trajectory group memberships (Supplementary Table S1).

The Supplementary Material for the STAGE cohort (Supplementary Table S2 and Supplementary Figures S3 and S4) show that the four-trajectory-group model was the best for those with night work, whereas those without night work had three trajectory groups. However, the stable sustainable working life trajectory group was largest (81.4–83.2%) in both.

For the STAGE cohort, the crude measure of night work (“yes”) was associated with both decreasingly and the increasingly sustainable working life, while the history of night work (1–10 years) was associated with a decreased risk of belonging to the same trajectory groups (Table 4) in comparison with the trajectory group of sustainable working life. Each one-year increase in age was associated with a higher likelihood of belonging to the decreasingly sustainable working life or stable lack of sustainable working life trajectory groups, but with less likelihood of an increasingly sustainable working life. Being a woman (compared to being a man) was associated with a higher likelihood of belonging to the decreasingly or increasingly sustainable working life group in comparison to the stable sustainable working life group. Being married (vs. not being married) and having higher education were associated with a lower likelihood of all group memberships in comparison with the stable sustainable working life trajectory group. Working in the private sector was only associated with a decreasingly sustainable working life. 

The analysis of discordant MZ and DZ twins involved in night work in the STAGE cohort showed that the effect of the occupational group on the likelihood of belonging to the trajectory groups might have had some familial confounding, since associations were seen in DZ twins, but not MZ twins (Supplementary Table S2). Other likelihoods for trajectory group membership were like those in the whole cohort.

## 5. Discussion

This prospective study of Swedish twins utilized two cohorts with relatively large sample sizes to investigate the trajectories of sustainable working life over 10–13 years and the effects of baseline night work on trajectory group memberships. For the older age group (SALT cohort), five trajectory groups were identified: stable sustainable working life (39.6%), stable lack of sustainable working life (24.8%), later decreasingly sustainable working life (15.0%), increasingly sustainable working life (13.6%), and early decreasingly sustainable working life (6.9%). The number of trajectories was four in the younger age group (STAGE cohort); these included stable sustainable working life (82.6%), decreasingly sustainable working life (7.0%), stable lack of sustainable working life (5.3%), and increasingly sustainable working life (5.1%). Regardless of whether it was measured as having participated or as the number of years of night work, night work was associated with a lower risk of belonging to the later decreasingly sustainable working life and increasingly sustainable working life trajectory groups in comparison to the stable sustainable working life group in the older cohort (SALT). Instead, in the younger (STAGE) cohort, the crude measure of night work (yes) was associated with both a decreasingly and increasingly sustainable working life, while a history of night work (1–10 years) was associated with a decreased risk of belonging to the same trajectory groups as those with the crude measure. These results add to the earlier knowledge based on studies of SA/DP [34,35,36], but also point towards the need to assess work factors other than only night work for the promotion of a sustainable working life. To the best of our knowledge, this study might be among the first to indicate the longitudinal development of working life trajectories and the predictive role of night work for them.

The observed trajectory of stable sustainable working life was the most common in both cohorts of different ages, which adds to the findings of earlier research, which has rarely investigated being at work, since the focus has been on SA or DP. However, our finding of a stable lack of a sustainable working life (around 5% in both cohorts) is also in line with earlier studies, since both short- and long-term work incapacity in terms of SA/DP are prevalent in the population [34,35,36]. However, the trajectory groups remained the same among those both with and without baseline night work. The hypothesis was that those with night work might have had low or a lack of a sustainable working life, since night work was previously linked with impaired health and with SA/DP [5,12,13,14]. Furthermore, night work was also expected to play a role in sustainable working life due to known mechanisms that function via circadian dysrhythmia and disturbed sleep [2,15]. Instead, we observed mixed effects of night work. In the older cohort (SALT), night work implied increased and decreased risk for different trajectories from those among the younger cohort (STAGE). Hence, one can assume that factors other than night work may have a greater influence on sustainable working life. Hence, other work-related aspects might be better targets for support or modifications at workplaces, as would individual factors, i.e., the employee and his/her characteristics.

Based on the trajectories of the two cohorts with different ages in this study, a higher age was associated with belonging to all trajectory groups in both cohorts, which is in line with earlier studies on shift work [26,27]. Since the directions of associations were opposite between the older (SALT) and younger (STAGE) cohorts, this might reflect two aspects. First, the baseline health and/or workability of these two cohorts might have been different. Second, the “healthy worker effect” may play a role, i.e., those who remain in working life at older ages are healthier than those who drop out. However, this should be confirmed and further studied in other studies with a special focus. Although the numbers of trajectory groups differed between the two cohorts, the trajectory of a later decreasingly sustainable working life over time in the SALT cohort was probably a consequence of the individuals being older both at the baseline and at the end of the follow-up. Furthermore, we were not able to detect a “healthy worker effect”, i.e., where those who did not tolerate or that had difficulties with night work did not continue, but transferred to day work [22], since in both cohorts, the trajectories were similar among those with or without night work (Supplementary Figures S1–S4). However, one can assume that the associations of night work and health [24,25] might play a role, and studies with the possibility of evaluating both health and night work together with working life statuses would be merited. Among the studied factors for trajectory group membership, higher education predicted a decreased likelihood for not having a sustainable working life for both cohorts. This finding aligns with studies of SA/DP [12,13,19].

The strengths of our study included the focus on sustainable working life, which added to the earlier results on the “lack of sustainability” part of working life (i.e., SA/DP) [12,13,14,30]. Furthermore, the twin sample enabled us to study the effects of familial confounding on associations with working life trajectories, which had not been done before [12,13,31]. In the older (SALT) cohort, we detected no effects of familial confounding, but in the younger (STAGE) cohort, familial confounding seemed to play a role in trajectory group membership and especially in the associations of the occupational sector and education with the trajectory groups among discordant twin pairs involved in night work. Furthermore, access to comprehensive national registry data provided strength to this study, as these data included tens of thousands of individuals with no loss in follow-up or recall bias for sustainable working life, and various influential factors were included in the regression models. Although we had detailed data, including dates for SA and DP, we lacked the data for short-term SA (<14 days). In addition, the assessment of night work relied on survey data and, therefore, had a limited level of detail. Night work was assessed with only one question, which was not validated, and we cannot rule out recall bias, and the interpretations of the respondents concerning the meaning of “worked nights” may have played a role. However, despite these limitations, the question provided a proxy for night work at baseline, and gathering data from large samples by other means than surveys is complicated. Still, the results might point towards an assumption that attention to the individual level and to work-related aspects other than night work could potentially support a sustainable working life. This should be confirmed in further studies. Another limitation may be related to the generalizability of our results. The assessment of sustainable working life was based on the welfare system in Sweden; therefore, the results might apply to other Nordic countries with similar welfare systems, but less so to other countries. 

## 6. Conclusions

Stable sustainable working life was the largest trajectory group in both cohorts, and a minor trajectory group had a stable lack of a sustainable working life. Night work played a mixed role in belonging to both the decreasingly and increasingly sustainable working life trajectory groups in both cohorts. Sociodemographic factors had an influence on the trajectories of sustainable working lives. 

## Figures and Tables

**Table 1:** Descriptive characteristics of the SALT and STAGE cohorts in this study.

	SALT (n = 34,680)		STAGE (n = 19,637)	
	n	%	n	%
Sex (women)	18,405	53	10,932	56
Marital status (married or living with someone)	21,608	62	7507	38
Education				
0–9 years	10,036	30	1165	6
10–12 years	14,968	44	9461	48
>12 years	8988	26	9002	46
Occupational sector				
Private sector	12,644	36	10,206	38
Public sector	10,815	31	6587	58
Other	1715	5	743	4
Missing	9506	27	-	-
Night work				
Yes	10,697	31	6606	34
No	23,983	69	12,689	65
History of night work				
None	23,983	69	12,689	66
1–10 years	6484	19	3975	30
>10 years	4213	12	721	4

**Table 2:** Goodness-of-fit statistics of the group-based trajectory analysis models.

	SALT				
	Smallest Group		BIC	AIC	APP
	N	%			
**2-cluster model**	**9494**	**36**	−92,323.86	−92,303.49	0.97
3-cluster model	6590	26	−82,611.44	−82,578.70	0.89
4-cluster model	1066	7	−79,690.03	−79,645.00	0.89
5-cluster model *	**1235**	**7**	**−76,517.72**	**−76,460.41**	**0.88**
6-cluster model	698	4	−75,727.56	−75,657.97	0.85
7-cluster model	727	5	−74,781.76	−74,699.89	0.87
8-cluster model	561	4	−74,165.62	−74,071.47	0.86
9-cluster model	538	3	−73,699.82	−73,593.40	0.82
	**STAGE**				
2-cluster model	2165	11	−38,424.04	−38,404.38	0.97
3-cluster model	1208	6	−35,474.10	−35,442.64	0.96
4-cluster model *	**927**	**5**	**−34,746.99**	**−34,703.73**	**0.84**
5-cluster model	581	3	−34,048.57	−33,993.51	0.87
6-cluster model	455	3	−33,906.68	−33,839.82	0.92

* The models presented are shown in bold. BIC = Bayesian information criterion, AIC = Akaike information criterion, and APP = average posterior probability.

**Table 3:** Relative-risk ratios (RRs) with 95% confidence intervals (Cis) for sociodemographic factors in relation to sustainable working life trajectory groups in the SALT cohort (with trajectory group 1 (sustainable working life) as a reference).

	SALT							
	Trajectory Group 2: Stable Lack of Sustainable Working Life		Trajectory Group 3: Later Decreasingly Sustainable Working Life		Trajectory Group 4: Increasingly Sustainable Working Life		Trajectory Group 5: Early Decreasingly Sustainable Working Life	
	RR	95%CI	RR	95%CI	RR	95%CI	RR	95%CI
Age at baseline	**0.95**	**0.95, 0.96**	**0.93**	**0.93, 0.94**	**1.04**	**1.03, 1.04**	**0.87**	**0.86, 0.88**
Sex (women)	**1.17**	**1.10, 1.25**	**1.17**	**1.08, 1.28**	**1.27**	**1.17, 1.38**	**1.46**	**1.28, 1.66**
Marital status (married or living with someone)	**0.89**	**0.84, 0.95**	**1.17**	**1.07, 1.26**	**1.12**	**1.03, 1.21**	**0.83**	**0.73, 0.93**
Education								
0–9 years	1	ref	1	ref	1	ref	1	ref
10–12 years	1.04	0.97, 1.11	**1.12**	**1.01, 1.24**	1.02	0.93, 1.12	1.15	0.98, 1.34
>12 years	**0.57**	**0.52, 0.63**	**0.78**	**0.70, 0.88**	**0.64**	**0.58, 0.72**	0.86	0.72, 1.04
Occupational sector								
Public sector	1	ref	1	ref	1	ref	1	ref
Private sector	**1.22**	**1.12, 1.34**	**1.13**	**1.04, 1.24**	**1.12**	**1.03, 1.22**	**1.42**	**1.22, 1.64**
Other	**2.63**	**2.29, 3.02**	**0.45**	**0.36, 0.57**	**0.52**	**0.43, 0.63**	**1.73**	**1.32, 2.26**
Nightwork *								
No (reference)	1	ref	1	ref	1	ref	1	ref
Yes	1.03	0.97, 1.10	**0.79**	**0.73, 0.86**	**0.86**	**0.80, 0.94**	1.00	0.88, 1.14
History of night work *								
No (reference)	1	ref	1	ref	1	ref	1	ref
1–10 years	1.08	1.00, 1.16	**0.75**	**0.68, 0.84**	**0.88**	**0.80, 0.97**	1.06	0.91, 1.23
>10 years	0.96	0.87, 1.05	**0.85**	**0.76, 0.96**	**0.84**	**0.75, 0.94**	0.90	0.74, 1.10

* Nightwork as a binary of “yes/no” and history of night work were modeled separately. Statistically significant OR with 95%CI in bold.

**Table 4:** Relative-risk ratios (RRs) with 95% confidence intervals (CIs) for sociodemographic factors and night work in relation to the sustainable working life trajectory groups in the STAGE cohort (with trajectory group 1 (sustainable working life) as a reference).

	STAGE					
	Trajectory Group 2: Decreasingly Sustainable Working Life		Trajectory Group 3: Stable Lack of Sustainable Working Life		Trajectory Group 4: Increasingly Sustainable Working Life	
	RR	95%CI	RR	95%CI	RR	95%CI
Age at baseline	**1.02**	**1.01, 1.03**	**1.04**	**1.02, 1.06**	**0.98**	**0.96, 0.99**
Sex (women)	**1.42**	**1.25, 1.62**	1.15	0.92, 1.43	**1.36**	**1.14, 1.63**
Marital status (married or living with someone)	**0.69**	**0.60, 0.79**	**0.58**	**0.47, 0.72**	**0.64**	**0.53, 0.78**
Education						
0–9 years	1	ref	**1**	**ref**	**1**	**ref**
10–12 years	**0.63**	**0.50, 0.78**	**0.51**	**0.37, 0.70**	**0.68**	**0.50, 0.94**
>12 years	**0.34**	**0.27, 0.44**	**0.16**	**0.11, 0.24**	**0.41**	**0.29, 0.57**
Occupational sector						
Public sector	1	ref	1	ref	1	ref
Private sector	**1.47**	**1.28, 1.70**	0.85	0.67, 1.08	1.06	0.87, 1.28
Other	1.28	0.93, 1.78	**2.61**	**1.83, 3.72**	**1.89**	**1.34, 2.66**
Nightwork *						
No (reference)	1	ref	1	ref	1	ref
Yes	**1.21**	**1.06, 1.37**	1.08	0.87, 1.34	**1.20**	**1.01, 1.43**
History of night work *						
No (reference)	1	ref	1	ref	1	ref
1–10 years	**1.29**	**1.13, 1.47**	1.19	0.95, 1.48	**1.24**	**1.03, 1.48**
>10 years	0.73	0.51, 1.05	0.54	0.28, 1.04	0.95	0.59, 1.55

* Nightwork as a binary (“yes/no”) and history of night work were modeled separately. Statistically significant OR with 95%CI in bold.

## Data Availability

The data that support the findings of this study are available from the original sources: the Swedish Twin Registry, Statistics Sweden, the Swedish Social Insurance Agency, and the Swedish National Board of Health and Welfare. Restrictions apply to the availability of the data used in this study based on the Swedish Twin Project of Disability Pension and Sickness Absence (STODS), which were used with ethical permission for the current study and, therefore, are not publicly available.

## Supplementary Materials

The following supporting information can be downloaded at: https://www.mdpi.com/article/10.3390/ijerph191710857/s1, Table S1: Relative-risk ratios (RR) with 95% confidence intervals (CI) for sociodemographic factors and night work in relation to sustainable working life trajectory groups among night work discordant twin pairs in SALT cohort (Trajectory group 1: Sustainable working life as reference); Table S2: Relative-risk ratios (RR) with 95% confidence intervals (CI) for sociodemographic factors and night work in relation to sustainable working life trajectory groups among night work discordant twin pairs in STAGE-cohort (Trajectory group 1: Sustainable working life as reference); Table S3: Descriptive characteristics of SALT- and STAGE-cohorts across baseline nightwork status; Table S4: Goodness of fit statistics of group-based trajectory analysis models across baseline night work status; Figure S1: Those with baseline nightwork in SALT cohort; Figure S2: Those without baseline nightwork in SALT-cohort; Figure S3 Those with nightwork in STAGE-cohort; Figure S4: Those without nightwork in STAGE-cohort.
