# Supplementary material for: Night Work and Sustainable Working Life—A Prospective Trajectory Analysis of Swedish Twins
Source: Int J Environ Res Public Health. 2022 Aug 31;19(17):10857. doi: 10.3390/ijerph191710857 (PMC9518065; doi:10.3390/ijerph191710857)
Supplement: Supplementary file 1 [file ijerph-19-10857-s001.zip › ijerph-1795271-supplementary.pdf]

Supplemental Table S1 Relative-risk ratios (RR) with 95% confidence intervals (CI) for sociodemographic factors and night work in relation to sustainable working life trajectory groups among night work discordant twin pairs in SALT cohort (Trajectory group 1: Sustainable working life as reference)

	**SALT : night work discordant pairs**															
	**Trajectory group 2: stable lack of sustainable working life**				**Trajectory group 3: later decreasing sustainable working life**				**Trajectory group 4: increasing sustainable working life**				**Trajectory group 5: early decreasing sustainable working life**			
	**MZ**		**DZ**		**MZ**		**DZ**		**MZ**		**DZ**		**MZ**		**DZ**	
	**RR**	**95%CI**	**RR**	**95%CI**	**RR**	**95%CI**	**RR**	**95%CI**	**RR**	**95%CI**	**RR**	**95%CI**	**RR**	**95%CI**	**RR**	**95%CI**
Marital status (married or living with someone)	1.01	0.79, 1.29	1.02	0.85, 1.21	1.11	0.86, 1.44	1.01	0.84, 1.21	0.81	0.55, 1.20	0.95	0.72, 1.24	0.92	0.78, 1.10	0.96	0.84, 1.10
Education																
0-9 years	1	ref	1	ref	1	ref	1	ref	1	ref	1	ref	1	ref	1	ref
10-12 years	0.92	0.69, 1.22	1.15	0.92, 1.42	1.18	0.84, 1.65	1.06	0.84, 1.33	1.37	0.78, 2.43	1.14	0.81, 1.60	1.13	0.92, 1.40	1.13	0.97, 1.32
>12 years	**0.51**	**0.36, 0.72**	0.78	0.61, 1.00	0.77	0.52, 1.13	**0.72**	**0.55, 0.94**	1.11	0.59, 2.11	0.71	0.47, 1.08	**0.69**	**0.53, 0.90**	**0.57**	**0.46, 0.70**
Occupational sector																
Public sector	1	ref	1	ref	1	ref	1	ref	1	ref	1	ref	1	ref	1	ref
Private sector	1.15	0.88, 1.49	1.21	0.99, 1.48	1.13	0.85, 1.51	**1.28**	**1.04, 1.59**	**1.76**	**1.07, 2.90**	1.33	0.96, 1.83	1.23	0.94, 1.62	1.16	0.94, 1.44
Other	0.56	0.30, 1.04	**0.63**	**0.41, 0.98**	0.50	0.24, 1.13	**0.53**	**0.31, 0.90**	**4.31**	**2.09, 8.88**	1.38	0.72, 2.62	**2.39**	**1.56, 3.67**	**2.84**	**2.08, 3.87**
Nightwork*																
No (reference)	1	ref	1	ref	1	ref	1	ref	1	ref	1	ref	1	ref	1	ref
Yes	0.90	0.72, 1.11	0.93	0.79, 1.10	0.84	0.67, 1.05	**0.78**	**0.66, 0.93**	0.88	0.59, 1.30	0.93	0.71, 1.22	0.94	0.81, 1.08	0.99	0.88, 1.11
History of night work*																
No (reference)	1	ref	1	ref	1	ref	1	ref	1	ref	1	ref	1	ref	1	ref
1-10 years	1.02	0.80, 1.31	0.95	0.78, 1.15	0.75	0.57, 1.00	**0.76**	**0.61, 0.92**	0.92	0.59, 1.43	0.91	0.67, 1.25	1.00	0.85, 1.18	1.04	0.91, 1.19
>10 years	**0.69**	**0.49, 0.97**	0.90	0.71, 1.14	0.99	0.72, 1.35	0.83	0.65, 1.06	0.81	0.44, 1.48	0.96	0.66, 1.40	0.82	0.65, 1.04	0.90	0.75, 1.07

Supplemental Table S2 Relative-risk ratios (RR) with 95% confidence intervals (CI) for sociodemographic factors and night work in relation to sustainable working life trajectory groups among night work discordant twin pairs in STAGE-cohort (Trajectory group 1: Sustainable working life as reference)

	**STAGE: night work discordant twin pairs**											
	**Trajectory group 2: decreasing sustainable working life**				**Trajectory group 3: stable lack of sustainable working life**				**Trajectory group 4: increasing sustainable working life**			
	**MZ**		**DZ**		**MZ**		**DZ**		**MZ**		**DZ**	
	**RR**	**95%CI**	**RR**	**95%CI**	**RR**	**95%CI**	**RR**	**95%CI**	**RR**	**95%CI**	**RR**	**95%CI**
Marital status (married or living with someone)	0.79	0.49, 1.29	0.71	0.44, 1,14	0.89	0.42, 1.90	0.53	0.19, 1.43	0.64	0.34, 1.23	**0.44**	**0.20, 0.98**
Education												
0-9 years	1	ref	1	ref	1	ref	1	ref	1	ref	1	ref
10-12 years	0.88	0.36, 2.17	**0.50**	**0.27, 0.94**	1.39	0.31, 6.17	0.54	0.11, 2.58	2.97	0.39, 22.64	0.64	0.22, 1.89
>12 years	**0.37**	**0.14, 0.99**	**0.22**	**0.11, 0.47**	0.46	0.10, 2.17	0.42	0.08, 2.30	3.11	0.40, 24.05	0.41	0.13, 1.29
Occupational sector												
Public sector	1	ref	1	ref	1	ref	1	ref	1	ref	1	ref
Private sector	0.92	0.56, 1.52	**2.55**	**1.51, 4.30**	1.12	0.49, 2.56	1.04	0.40, 2.70	**1.88**	**1.00, 3.52**	1.37	0.65, 2.90
Other	0.64	0.14, 2.96	2.79	0.93, 8.36	**5.65**	**1.79, 17.79**	na	-	1.53	0.33, 24.05	3.55	0.96, 13.15
Nightwork*												
No (reference)	1	ref	1	ref	1	ref	1	ref	1	ref	1	ref
Yes	1.02	0.77, 1.35	1.22	0.92, 1.61	0.98	0.62, 1.53	0.64	0.36, 1.15	0.97	0.68, 1.38	0.83	0.55, 1.24
History of night work*												
No (reference)	1	ref	1	ref	1	ref	1	ref	1	ref	1	ref
1-10 years	1.26	0.76, 2.08	0.94	0.58, 1.54	0.80	0.39, 1.64	0.46	0.19, 1.14	0.88	0.49, 1.57	0.90	0.46, 1.75
>10 years	0.51	0.16, 1.56	0.52	0.22, 1.23	0.40	0.09, 1.76	0.16	0.02, 1.38	0.86	0.28, 2.66	0.63	0.17, 2.27

na = not able to be assessed due to low number of observations

Supplemental Table S3 Descriptive characteristics of SALT- and STAGE-cohorts across baseline nightwork status

	SALT				STAGE			
	Nightwork (n = 10 697)		No nightwork (n = 23 983)		Nightwork (n= 6 606)		No nightwork (n = 12 689)	
	**Mean**	**SD**	**Mean**	**SD**	**Mean**	**SD**	**Mean**	**SD**
Age at baseline	58.4	8.0	59.3	7.9	37.3	7.2	37.4	7.0
	**n**	**%**	**n**	**%**	**n**	**%**	**n**	**%**
Sex (women)	4448	42	13957	58	7501	59	3252	49
Marital status (married or living with someone)	6390	60	15218	63	2325	35	5090	40
Education								
0-9 years	2865	27	7171	31	450	7	695	5
10-12 years	4270	45	10248	44	3365	51	5917	47
>12 years	2925	28	6063	26	2789	42	6070	48
Occupational sector								
Private sector	3668	35	8894	37	3185	54	6853	60
Public sector	3750	34	7147	30	2516	43	3962	35
Other	444	4	1271	5	195	3	538	5
Missing	2835	27	6671	28	-	-	-	-

Supplemental Table S4 Goodness of fit statistics of group-based trajectory analysis models across baseline night work status

	SALT									
	Nightwork					No nightwork				
	Smallest group		BIC	AIC	APP	Smallest group		BIC	AIC	APP
	N	%				N	%			
2-cluster model	2998	36	-29087.2	-29069.6	0.97	6506	36	-63214.8	-63195.3	0.97
3-cluster model	2316	24	-25844.2	-25816.1	0.96	4686	28	-56768.4	56737.1	0.89
4-cluster model	394	7	-24889.8	-24851.1	0.85	696	6	-54805.2	-54762.2	0.90
**5-cluster model***	**398**	**7**	**-24035.4**	**-23986.2**	**0.89**	**836**	**7**	**-52490.7**	**-52436.1**	**0.92**
6-cluster model	463	5	-23831.2	-23771.5	0.89	423	4	-51896.6	-51830.3	0.88
7-cluster model	258	5	-23496.0	-23425.7	0.86	145	3	-51826.1	-51748.1	0.86
8-cluster model	135	3	-23457.3	-23376.5	0.78	-	0	-51022.0	-50932.2	-
	STAGE									
2-cluster model	784	12	-13662.0	-13645.1	0.99	1334	11	-23937.3	-23918.7	0.97
3-cluster model	430	7	-12703.5	-12676.4	0.97	745	6	-22002.6	-21972.9	0.96
**4-cluster model***	**346**	**5**	**-12369.8**	**-12332.6**	**0.85**	**621**	**5**	**-21301.7**	**-21249.8**	**0.89**
5-cluster model	215	3	-12213.0	-12165.6	0.91	346	3	-21216.1	-21164.1	0.87
6-cluster model	111	2	-12158.7	-12101.1	0.86	180	1	-20968.9	-20905.8	0.84

* The models presented are shown in bold. BIC = Bayesian Information Criterion, AIC = Akaike Information Criterion, and APP = average posterior probability.

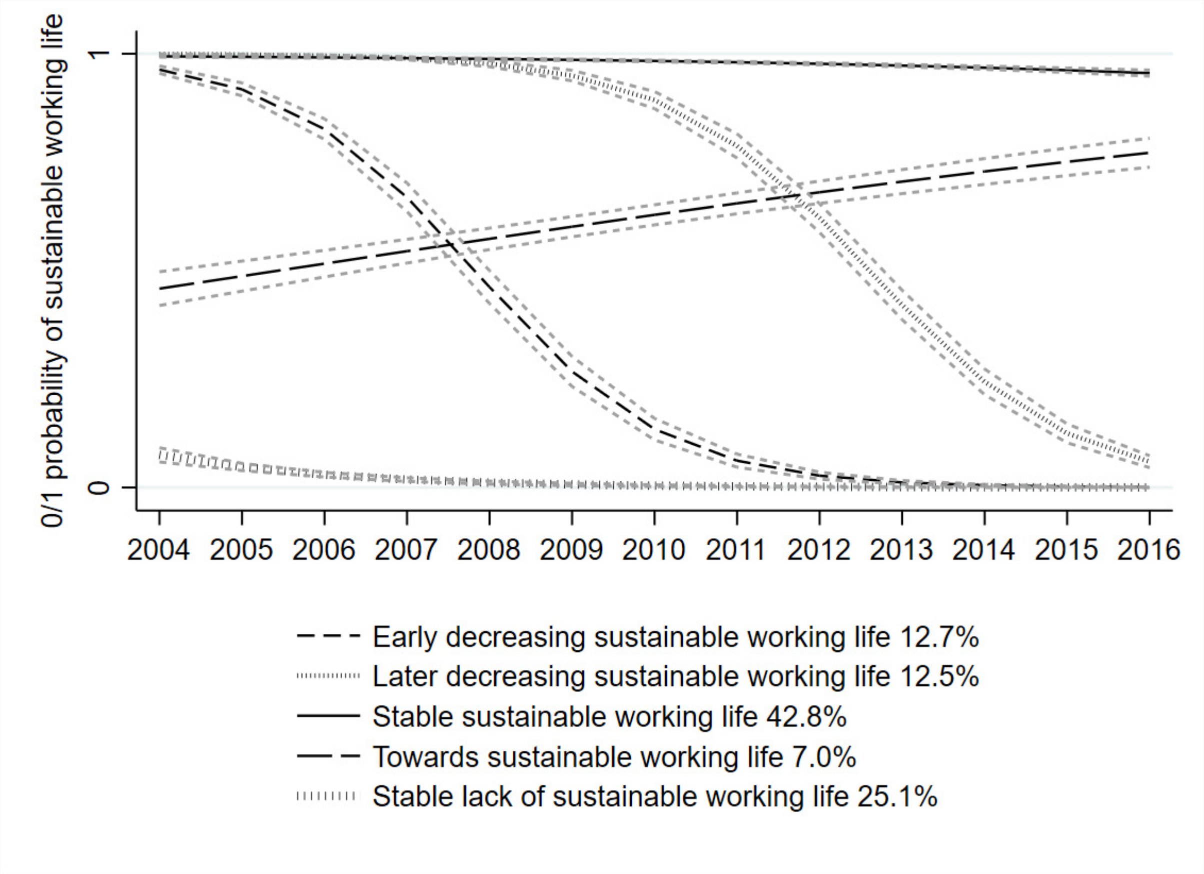

Supplemental Figure S1 Those with baseline nightwork in SALT cohort

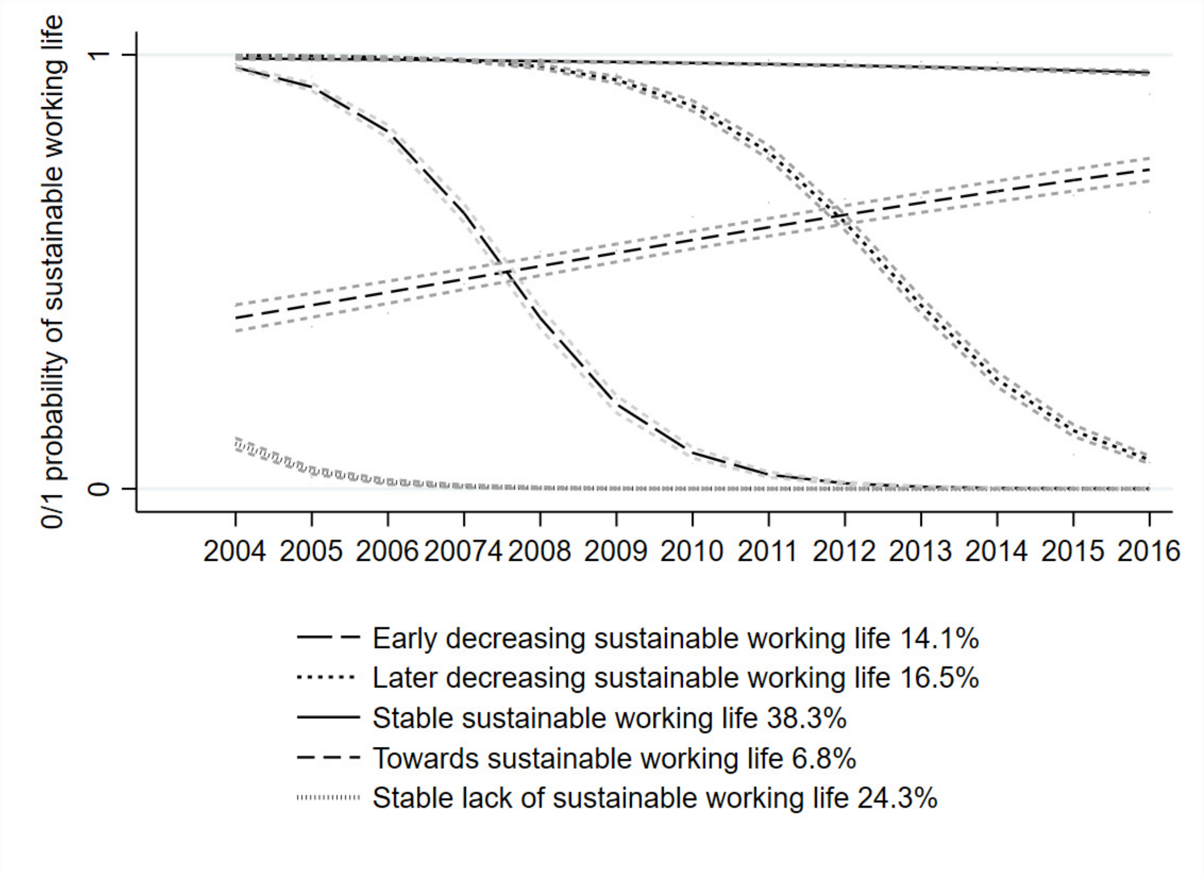

Supplemental Figure S2 Those without baseline nightwork in SALT-cohort

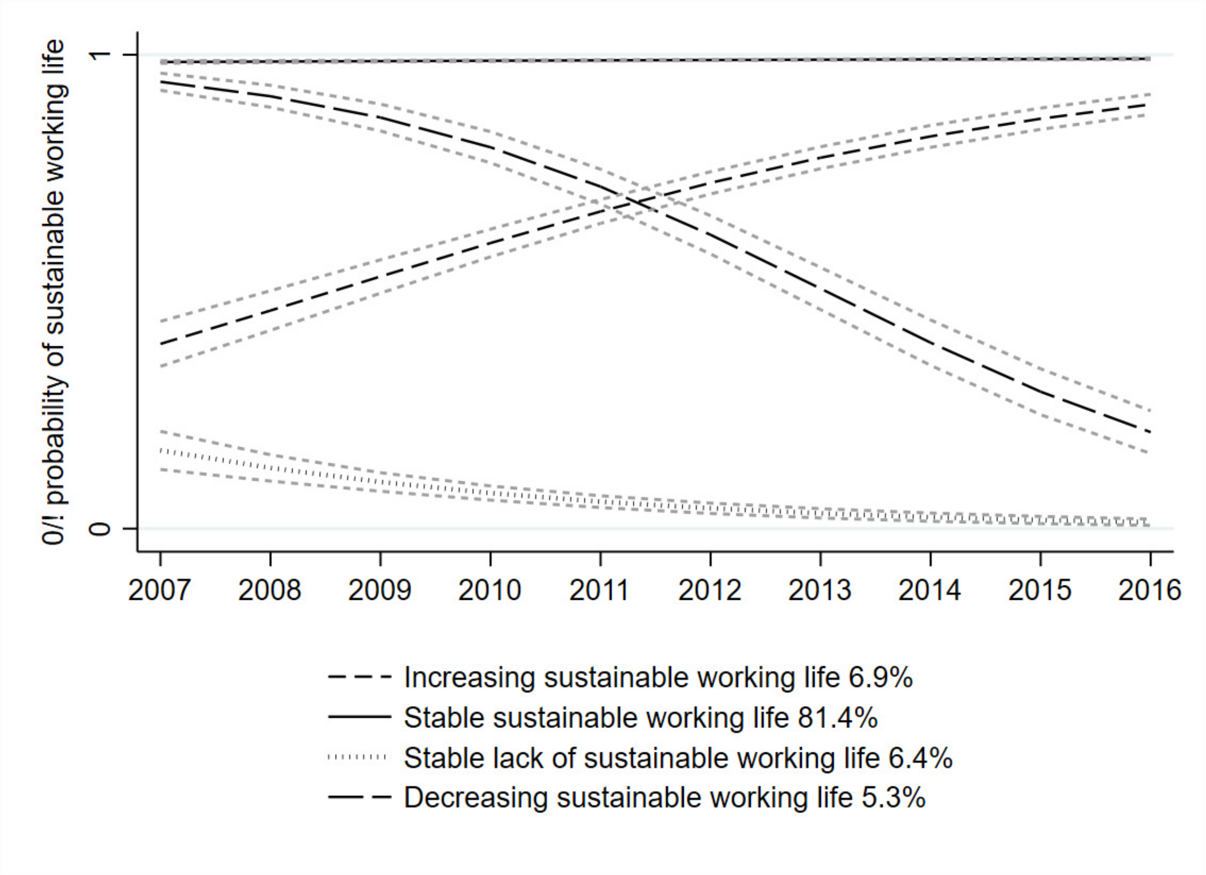

Supplemental Figure S3 Those with nightwork in STAGE-cohort

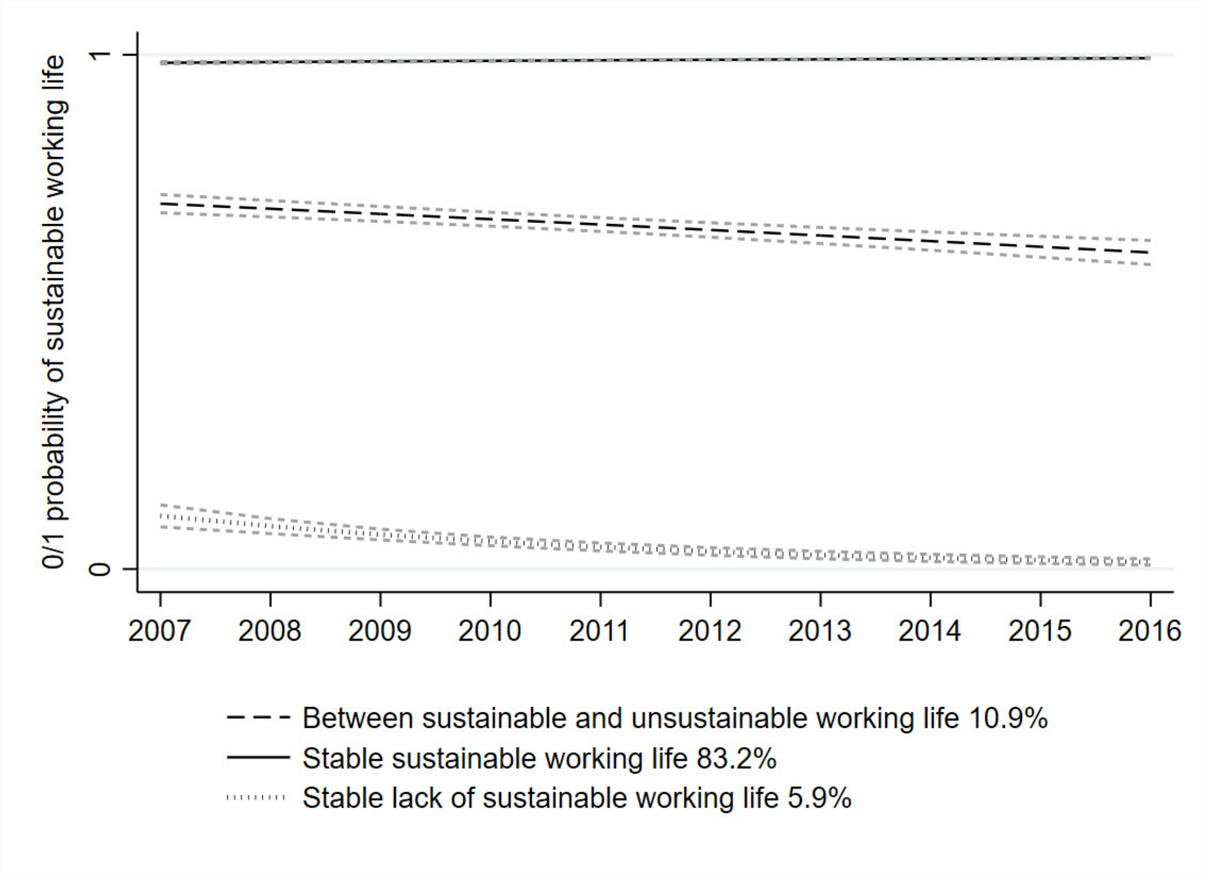

Supplemental Figure S4 Those without nightwork in STAGE-cohort
